# Distribution patterns of dinoflagellate communities along the Songhua River

**DOI:** 10.7717/peerj.6733

**Published:** 2019-04-09

**Authors:** Yangchun Gao, Yiyong Chen, Wei Xiong, Shiguo Li, Aibin Zhan

**Affiliations:** 1 Research Center for Eco-Environmental Sciences, Chinese Academy of Sciences, Beijing, China; 2 University of Chinese Academy of Sciences, Chinese Academy of Sciences, Beijing, China

**Keywords:** Metacommunity metabarcoding, Dinoflagellate, RDA, Species sorting, Dispersal

## Abstract

**Background:**

Dinoflagellates have the potential to pose severe ecological and economic damages to aquatic ecosystems. It is therefore largely needed to understand the causes and consequences of distribution patterns of dinoflagellate communities in order to manage potential environmental problems. However, a majority of studies have focused on marine ecosystems, while the geographical distribution patterns of dinoflagellate communities and associated determinants in freshwater ecosystems remain unexplored, particularly in running water ecosystems such as rivers and streams.

**Methods:**

Here we utilized multiple linear regression analysis and combined information on species composition recovered by high-throughput sequencing and spatial and environmental variables to analyze the distribution patterns of dinoflagellate communities along the Songhua River.

**Results:**

After high-throughput sequencing, a total of 490 operational taxonomic units (OTUs) were assigned to dinoflagellates, covering seven orders, 13 families and 22 genera. Although the sample sites were grouped into three distinctive clusters with significant difference (*p* < 0.05) in environmental variables, OTUs-based dinoflagellate communities among the three clusters showed no significant difference (*p* > 0.05). Among all 24 environmental factors, two environmental variables, including NO_3_-N and total dissolved solids (TDS), were selected as the significantly influential factors (*p* < 0.05) on the distribution patterns of dinoflagellate communities based on forward selection. The redundancy analysis (RDA) model showed that only a small proportion of community variation (6.1%) could be explained by both environmental (NO_3_-N and TDS) and dispersal predictors (watercourse distance) along the River. Variance partitioning revealed a larger contribution of local environmental factors (5.85%) than dispersal (0.50%) to the total variation of dinoflagellate communities.

**Discussion:**

Our findings indicated that in addition to the two quantifiable processes in this study (species sorting and dispersal), more unquantifiable stochastic processes such as temporal extinction and colonization events due to rainfall may be responsible for the observed geographical distribution of the dinoflagellate community along the Songhua River. Results obtained in this study suggested that deeper investigations covering different seasons are needed to understand the causes and consequences of geographical distribution patterns of dinoflagellate biodiversity in river ecosystems.

## Introduction

Aquatic ecosystems, such as rivers and lakes, support various habitats for diverse biological communities. Among those diverse communities, dinoflagellates (division Pyrrhophyta, class Dinophyceae) are ecologically important members of phytoplankton, as they play important roles in primary production, carbon cycling and oxygen release in aquatic ecosystems (Aufdenkampe et al., 2011; Gaines & Elbrächter, 1987). However, they can also pose serious economic and ecological damages to aquatic ecosystems, since some dinoflagellate species propagate quickly and form algal blooms under suitable environmental conditions. Dinoflagellate blooms largely threaten aquatic ecosystems as they cause significantly negative effects such as water fouling, oxygen deficiency, and large-scale mortality of species (Gao et al., 2017; Granéli & Turner, 2006). Hence, a deeper understanding of dinoflagellate assembly and their geographical distribution patterns is not only beneficial to the protection and management of aquatic ecosystems and associated industries such as fisheries and aquaculture, but also provides baseline information to effectively manage and conserve aquatic biodiversity (Altermatt, 2013; Heino, 2013).

Increasing evidence suggests that multiple factors can influence dinoflagellate community assembly and geographical distribution (Aydin et al., 2015; Gao et al., 2018; Liu et al., 2012). Major factors include environmental filtering (e.g., species sorting: species only occur at favorable environments), dispersal (e.g., mass effect: the strong mobility of individuals to reach close geographic sites; dispersal limitation: the limited ability of individuals to reach distant geographic sites) and stochastic processes (e.g., colonization and extinction). At different geographical scales, two major competing factors, dispersal and species sorting, have been commonly considered as fundamental processes in structuring biological communities in aquatic ecosystems (Beisner et al., 2006; Devercelli et al., 2016; Heino et al., 2015; Isabwe et al., 2018; Xiong et al., 2016; Xiong et al., 2017; Wu et al., 2017). In addition to the two major competing factors, other factors such as contrasting hydrographic conditions (e.g., wet season versus dry season) may also affect the spatial structure of aquatic communities (Isabwe et al., 2018). During wet seasons, frequent rainfall is expected to result in the change of connectivity among sample sites (Larned et al., 2010). Thus, temporal colonization and extinction events may easily occur due to the change of dispersal patterns of aquatic communities, especially for plankton communities (Heino et al., 2015; Larned et al., 2010). Such stochastic processes may largely contribute to the variation of plankton communities (Grönros et al., 2013; Heino et al., 2015). Given the complexity of various influential factors, the geographical distribution patterns of communities may largely vary among aquatic ecosystems. Although geographical variation of dinoflagellate communities and associated mechanisms have been well investigated in marine ecosystems (Ignatiades, 2012; Le Bescot et al., 2016; Gao et al., 2018; Granéli & Turner, 2006; Jeong et al., 2010), relevant reports in freshwater ecosystems such as rivers and streams are relatively rare.

In this study, dinoflagellate communities were collected along the Songhua River, which is located in northeast China. The Songhua River is the fifth longest river in China (1,927 km), and currently suffers from severe environmental stresses, mainly owing to increasing disturbance derived from human activities. Mountain regions were mainly polluted by heavy metals from gold mining and metal smelting (Zou et al., 2010). Urban regions were polluted by organic pollutions mainly discharged by domestic sewage (Wang et al., 2018), and these regions were often characterized by high concentrations of chemical oxygen demand (Lin et al., 2014). Severe nitrogenous and phosphorus pollution derived from non-point pollution of agriculture was severely problematic in rural regions (Yu et al., 2003). Here we collected dinoflagellate communities from different regions of the Songhua River, and all collected communities were profiled by metabarcoding, a powerful tool for characterizing microscopic communities (Abad et al., 2016; Gao et al., 2018; Xiong et al., 2017; Zhan et al., 2013). We combined information on species composition, as well as spatial and environmental variables, to analyze the community structure and geographical distribution of dinoflagellates during the wet season of 2017. We aimed to (I) characterize the spatial distribution patterns of dinoflagellate communities and (II) disentangle the main processes that largely influence geographical distribution of dinoflagellate biodiversity.

## Material and Method

### Sampling and DNA extraction

**Figure 1:**
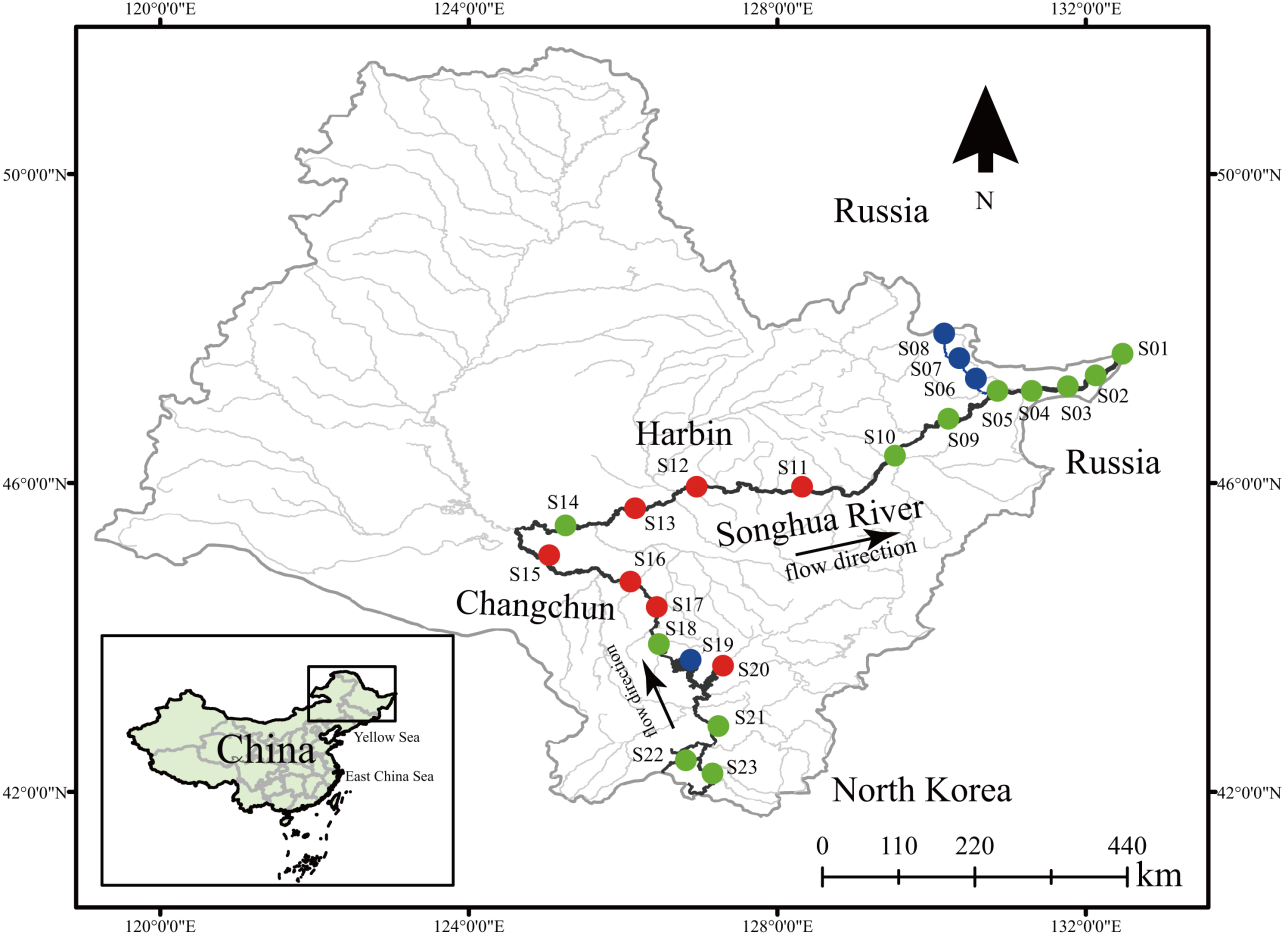
Sample locations of dinoflagellate communities along the Songhua River located in Northeast China. Dots in blue, red, and green represent sampling sites in Groups I, II, and III, respectively. All maps are made by ArcGIS version 10.0 (ESRI Company).

Dinoflagellate communities were sampled from 23 locations in July (wet season) of 2017. The sampling locations were relatively evenly distributed along the Songhua River (Fig. 1). For each sampling location, 30 l surface water (0–0.5 m) was collected and then filtered through a 25 µm mesh, and all residuals including dinoflagellates on the mesh were transferred into a 100 ml bottle and fixed with 100% alcohol for downstream analyses (Xiong et al., 2017). Meanwhile, 500 ml surface water was also collected and stored at 4 °C for measurement of environmental factors. Total genomic DNA of each dinoflagellate community was extracted using the DNeasy Blood and Tissue Kit (Qiagen Canada Inc., Toronto, ON, Canada). The quality of DNA was measured using NanoDrop 2000 (Thermo Scientific, Waltham, MA, USA).

### Metabarcoding

All dinoflagellate communities were characterized using a metabarcoding approach. The primer pair (Uni18S: AGGGCAAKYCTGGTGCCAGC; Uni18SR: GRCGGTATCTRATCGYCTT) (Zhan et al., 2013) was selected to characterize dinoflagellate communities. The pair of primers was designed based on V4 region of the 18S rDNA, and the length of amplicons varied from 400–600 bp among different species (Zhan et al., 2013). Although this pair of primers was originally designed for zooplankton, PCR amplification demonstrated that the pair was largely universal and could cover a wide range of aquatic taxa including dinoflagellates (Zhan et al., 2013; Zhan et al., 2014; Zhan & MacIsaac, 2015). In addition, the high universality of this primer pair for dinoflagellates was also confirmed by aligning representative dinoflagellate 18S with the primer pair (Fig. S1). Three PCR replicates for each sample were performed to avoid biased amplification (Zhan et al., 2013; Zhan et al., 2014; Zhan & MacIsaac, 2015). The PCR mixture (25 µl) consisted of 1 × *Taq* Buffer (with 20 mM Mg^2+^; Takara, Dalian, China), 5.0 mM of each dNTP, 10 pmol of forward primer with sample-specific tags and reverse primer, 0.5 U of TaKaRa *Taq* (Takara) and 100 ng of genomic DNA. PCRs were performed on a Mastercycler nexus (Eppendorf, Hamburg, Germany) with the following cycle conditions: 95 °C for 5 min; then 25 cycles at 95 °C for 30 s, 50 °C for 30 s and 72 °C for 30 s; and the final extension at 72 °C for 5 min. PCR products of the three replicates for each sample were pooled and purified using the SanPrep Spin PCR Products purification kit (Shanghai, China). Finally, a constructed sequencing library derived from the purified PCR products was sequenced using the Illumina Miseq PE300 sequencing Platform (Illumina, San Diego, CA, USA).

Raw sequence reads were denoised, trimmed and filtered using both USEARCH version 8.1 (Edgar, 2013) and RDP pipeline (https://pyro.cme.msu.edu/). Non-biological sequences (e.g., tags, primers and adapters) were removed using RDP. Subsequently, the expected error threshold of 0.5 was used to filter sequences with possible sequencing errors. Filtered sequences were de-replicated to obtain unique sequences, which were clustered into Operational Taxonomic Units (OTUs) at the 100% similarity (Le Bescot et al., 2016; Janouškovec et al., 2017). The obtained OTUs were annotated by searching against the Protist Ribosomal Reference database (PR2) (Guillou et al., 2013) using SEED version 1.46 (Větrovský & Baldrian, 2013) with the parameters of e value <10^−80^, minimum query coverage >95% and similarity >95%. OTUs assigned to non-dinoflagellate taxa were removed from our datasets. The relative proportion of each OTU was calculated in each sample and used as the proxy of relative OTU abundance for subsequent analyses (Hirai et al., 2015).

### Analyses of environmental factors

Environmental factors were analyzed according to the procedures described by Xiong et al. (2017). Briefly, water temperature (T), pH, oxidation–reduction potential (ORP), electrical conductivity (EC) and total dissolved solid (TDS) were measured *in situ* with a multiparameter sensor (MYRON company, USA). The chlorophyll-a (Chl_a) and dissolved oxygen (DO) were determined *in situ* with a Handheld Fluorometer (Turner Designs, San Jose, CA, USA) and a portable dissolved oxygen meter (HACH company, Loveland, CO, USA), respectively. Total nitrogen (TN), nitrate nitrogen (NO_3_-N) and ammonia nitrogen (NH_4_-N) were measured using the alkaline potassium persulfate digestion UV spectrophotometric method, ultraviolet spectrophotometry and Nessler’s reagent spectrophotometry, respectively. Total phosphorus (TP) and soluble reactive phosphorus (SRP) were determined based on the ammonium molybdate spectrophotometric method. Chemical oxygen demand (COD) and metals (K, Ca, Na, Mg, Cd, Cr, Cu, Ni, Zn, Pb, and As) were measured with HACH COD digestion vials (HACH Company, Loveland, CO, USA) and inductively coupled plasma-mass spectrometry (ICP-MS, 7500A, Plasma Quad 3, USA), respectively. A total of 24 environmental factors were collected in this study.

### Spatial variables

Dispersal, such as the mass effect in running rivers, often plays a key role in structuring plankton communities. To test the role of dispersal in shaping dinoflagellate communities, we measured the actual watercourse distance and used it for dispersal proxy between sampling sites (Beisner et al., 2006).

### Statistical analyses

Before statistical analyses, the relative abundance of OTUs and all measured environmental factors, except for pH, were log_10_ (*x* + 1) transformed to improve homoscedasticity. We first clustered sampling sites into distinctive groups based on the Euclidean distance of environmental variables using the CLUSTER program. To characterize distribution patterns of dinoflagellate communities, we performed further tests including an analysis of similarity (ANOSIM), an analysis of similarity percentages (SIMPER), and a nonmetric multidimensional scaling (NMDS) using non-parametric multivariate methods. The relative abundance of dinoflagellates between groups was compared using ANOSIM, which was based on Bray-Curtis distance and rank dissimilarity. The SIMPER analysis was used to identify the major OTUs responsible for the contribution of community variation at both the intra- and inter-group levels. An NMDS analysis on environmental variables was performed to profile the inter-group relativeness among sampling sites. Based on the NMDS results on environmental variables, we performed another NMDS analysis on dinoflagellate communities to primarily assess the potential influence of environmental factors on dinoflagellate communities. All CLUSTER, ANOSIM, SIMPER and NMDS analyses were performed in PRMIER 6.0 (Clarke & Gorley, 2001).

To disentangle the main processes that largely influence geographical distribution of dinoflagellate biodiversity, we performed a linear ordination method, redundancy analysis (RDA). Since collinearity among explanatory variables can lead to the inflation of type I error and overestimation of the amount of explained variation (Blanchet, Legendre & Borcard, 2008), we conducted forward selection to select significant environmental variables using the *forward.sel* function (ANOVAS; 1,000 permutations) implemented in *packfor* package in R (R Core Team, 2015). To demonstrate the relative contribution of species sorting and dispersal to the community structure variation, variance partitioning and partial redundancy analyses (pRDA) were performed to estimate the proportion of dinoflagellate community variation purely explained by environmental predictors (significant environmental factors) and dispersal (watercourse distance). Variance analyses (ANOVAS; 1,000 permutations) were performed to test the significance of RDA and pRDA. The analyses including RDA, variance partitioning and pRDA were computed using *vegan* package in R (R Core Team, 2015).

## Results

### Environmental factors

**Figure 2:**
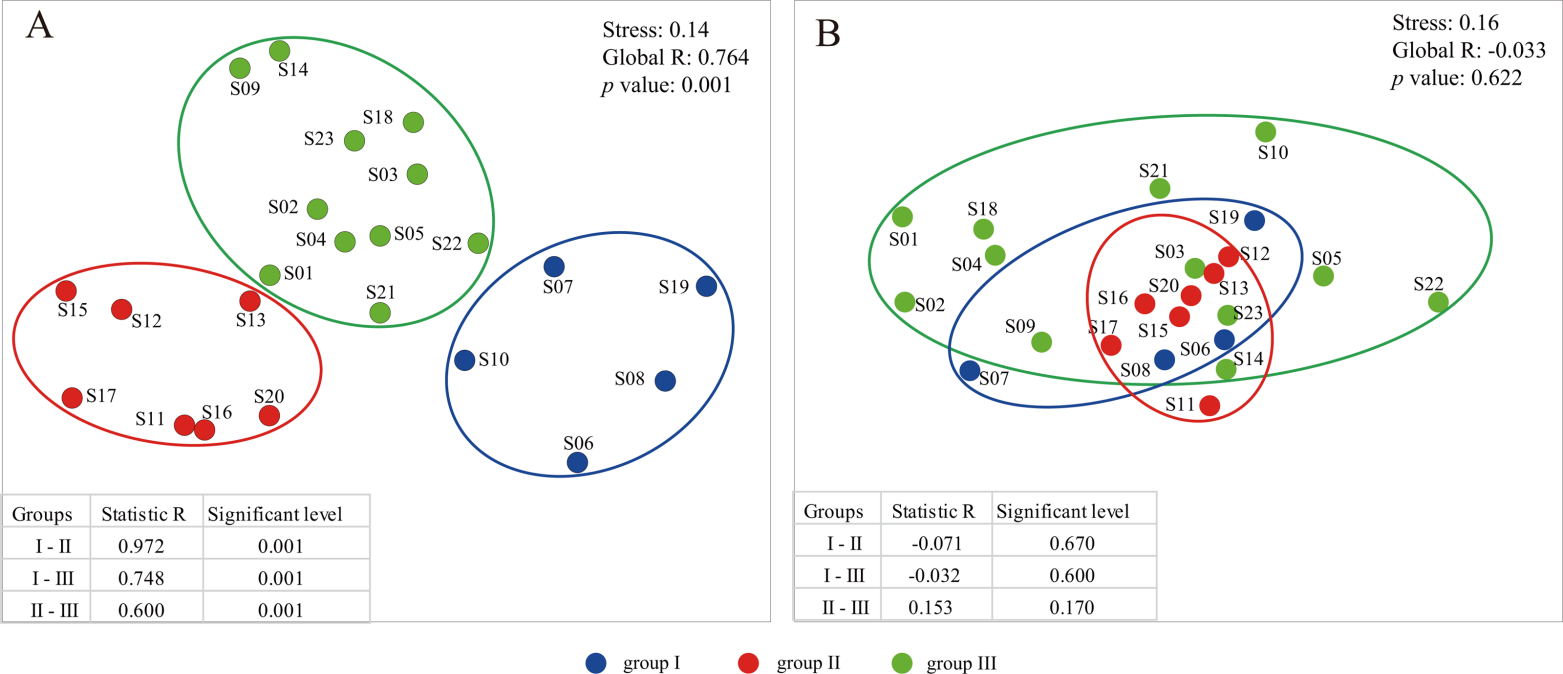
The plots of nonmetric multidimensional scaling ordination (NMDS) based on environmental variables (A) and dinoflagellate communities (B).

A total of 24 environmental factors were collected at the 23 sampling sites along the Songhua River (Table S1). Based on the environmental variables, all sampling sites were clustered into three distinctive groups (I, II, III) (Stress = 0.14; Figs. 1 and 2A). In addition, the ANOSIM analysis revealed significant differences in the environmental factors between the three groups (global *R* = 0.764; *p* = 0.001; Fig. 2A). The environmental factors largely varied among the three groups, particularly for NH_4_-N and NO_3_-N. The concentration of NH_4_-N was the lowest in the group I (0.01–0.24 mg l^−1^, mean = 0.074 mg l^−1^), higher in the group II (0.01–0.47 mg l^−1^, mean = 0.15 mg l^−1^), and the highest in the group III (0.01–2.74 mg l^−1^, mean = 0.66 mg l^−1^; Table S1). Similarly, NO_3_-N was the lowest in the group I (0.51–2.88 mg l^−1^, mean = 1.48 mg l^−1^) and the highest in the group III (0.42–11.61 mg l^−1^, mean = 6.24 mg l^−1^; Table S1).

### Composition and distribution of dinoflagellate communities

**Figure 3:**
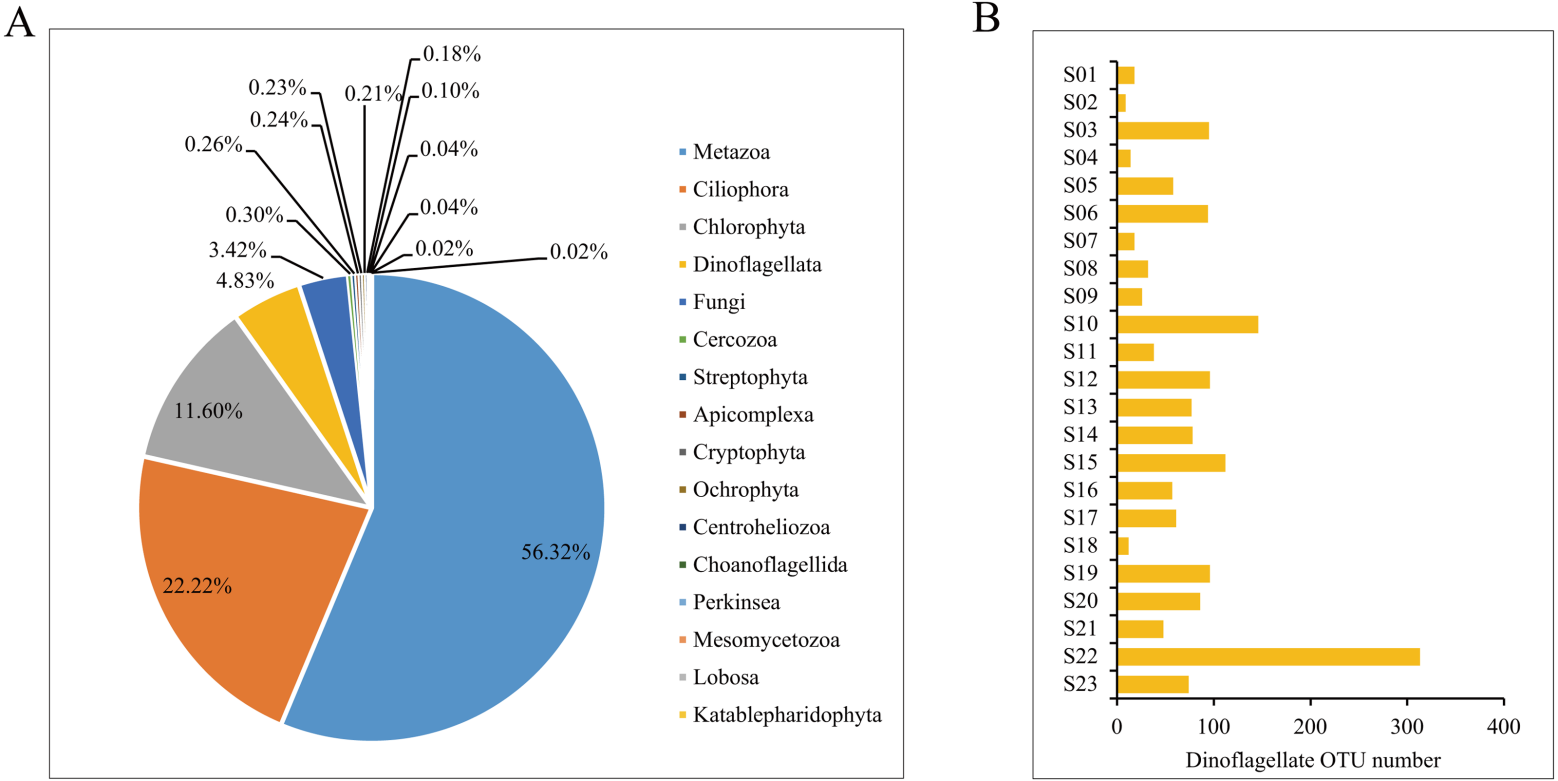
The annotation of Operational Taxonomic Units (OTUs). (A) Proportions of taxonomic composition; (B) the number of dinoflagellate OTUs per sample.

MiSeq sequencing produced a total of 346,490 raw reads from the 23 samples (NCBI SRA accession no. SRP151183). After quality filtering and OTU clustering at 100% similarity, a total of 11,530 OTUs were obtained, and the rarefaction curves for all samples reached saturation or almost saturation (Fig. S2), suggesting that the biodiversity was well recovered. Among the 11,530 OTUs, 10,153 OTUs were successfully annotated, covering Metazoa (56.32%), followed by Ciliophora (22.22%), Chlorophyta (11.60%) and Dinoflagellata (4.83%) (Fig. 3A). All 490 dinoflagellate OTUs were used for downstream analyses. The number of OTUs per sample site largely varied from 9 at site S02 to 313 at site S22, and the average number of OTUs per sample was 72 (Fig. 3B, Table S2).

**Figure 4:**
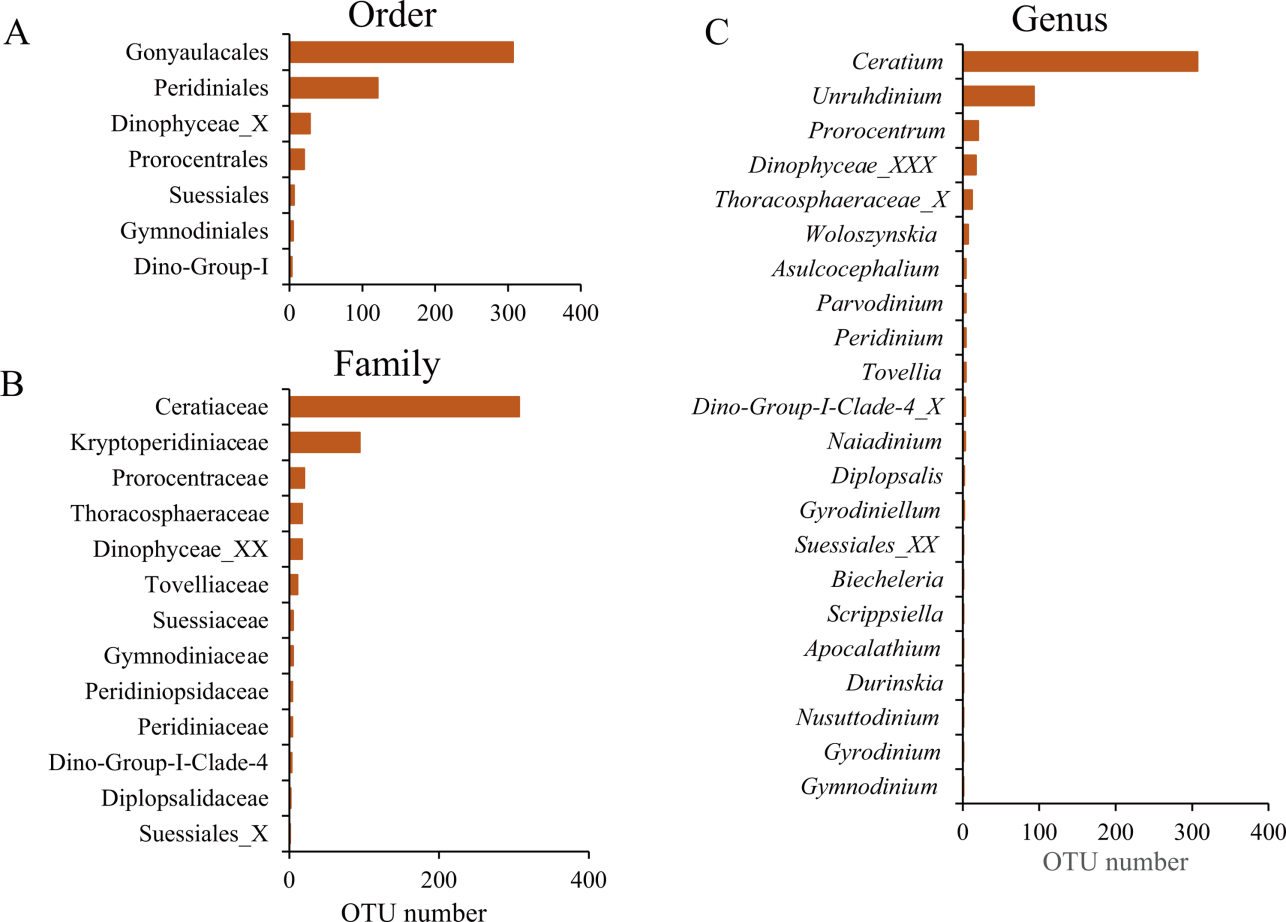
The Operational Taxonomic Unit (OTU) number of different taxonomic groups. (A) Order level; (B) Family level; (C) Genus level.

The taxonomic assignment of the dinoflagellate OTUs covered seven orders: Gonyaulacales, Peridiniales, Dinophyceae_X, Prorocentrales, Suessiales, Gymnodiniales and Dino-Group-I, of which Gonyaulacales was the most abundant taxa (307 OTUs), followed by Peridiniales (121 OTUs) and Dinophyceae_X (28 OTUs). From the seven orders, we identified 13 families, of which Ceratiaceae (307 OTUs), Kryptoperidiniaceae (94 OTUs) and Proroentraceae (20 OTUs) were identified as the first three dominant taxa. A total of 22 genera were retrieved, and the top six were *Ceratium* (307 OTUs), *Unruhdinium* (93 OTUs), *Prorocentrum* (20 OTUs), *Dinophyceae_XXX* (17 OTUs), *Thoracosphaeraceae_X* (12 OTUs) and *Woloszynskia* (7 OTUs) (Fig. 4, Table S2). Among the 490 OTUs, OTU_6 (Gouyaulacales, Ceratiaceae, *Ceratium*), OTU_7 (Peridiniales, Kryptoperidiniaceae, *Unruhdinium*) and OTU_115 (Peridiniales, Thoracosphaeraceae, *Thoracosphaeraceae_X*) were the top three taxa with highest relative abundance (Table S2).

To primarily assess the potential influence of environmental factors on distribution patterns of dinoflagellate communities, we also performed CLUSTER and NMDS analyses on dinoflagellate communities based on groups inferred from environmental variables. However, no significant variation was found among groups with a global R of −0.033 (*p* = 0.622; Fig. 2B), and the two clusters based on the dendrogram were not coincide with environmental groups (Fig. 2A; Fig. S3). In addition, we observed a high level of dissimilarity at the inter-group level; the average dissimilarity was 83.04 (*R* =  − 0.071, *p* = 0.670) between groups I and II, 77.94 (*R* =  − 0.032, *p* = 0.600) between groups I and III, and 68.91 (*R* = 0.153, *p* = 0.170) between groups II and III (Table S3; Fig. 2B). However, we found a low level of similarity at the intra-group level: the average similarity values were 12.39, 21.44 and 29.15 in groups I, II, and III, respectively (Table S3). In addition, the taxa responsible for the intra-group similarity were almost similar. For example, OTU_6 (Ceratiaceae, Ceratiaceae, *Ceratium*), which had wide geographical distribution and large variation in relative abundance among sample sites, contributed 54.74%, 83.44%, and 76.00% to the similarity within the groups I, II, III, respectively (Table S4). Meanwhile, the OTU_6 (Ceratiaceae, Ceratiaceae, *Ceratium*) was also one of the top contributors to the dissimilarity between groups, contributing 28.61%, 27.22%, and 28.12% to the dissimilarity between groups I and II, groups I and III, and groups II and III, respectively (Table S3).

### Influence of environmental factors on community structure

**Figure 5:**
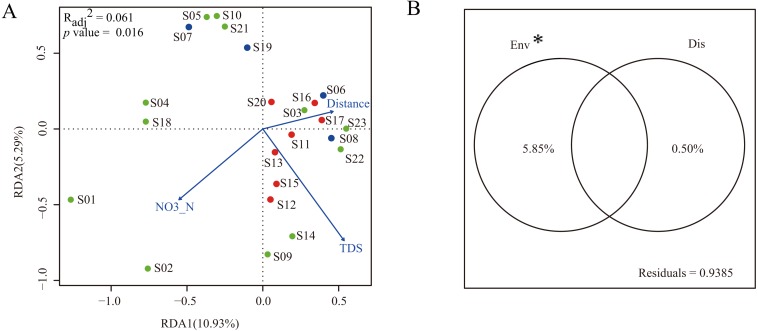
Multiple linear regress analysis. (A) The ordination plot based on redundancy analysis of dinoflagellate community with selected environmental predictor (NO_3_-N and TDS) and dispersal predictor (watercourse distance). Dots in blue, red, and green represent sampling sites in Groups I, II, and III, respectively; (B) the variance partitioning of the selected environmental predictors (NO_3_-N and TDS) and dispersal predictor (watercourse distance). “Env” and “Dis” represent environmental and dispersal predictors, respectively. “*” indicates significant difference (p < 0.05).

To construct a parsimonious RDA model, environmental factors with significant influence on dinoflagellate community structure were selected based on the results of forward selection. The results showed that two environmental factors (NO_3_-N and TDS) were selected and included in the RDA framework (Fig. 5A). The RDA was globally significant (*p* = 0.016) with an adjusted coefficient of determination (*R*_adj_^2^) of 0.061. The first two axes of the RDA model explained 10.93% and 5.29% of the total variation, respectively (Fig. 5A).

In order to characterize the relative contribution of environmental predictors (NO_3_-N and TDS) and spatial predictor (watercourse distance) to the observed dinoflagellate community structure, a variance partitioning was performed. The results demonstrated that the environmental predictors, which explained 5.85% of the total variation, had a significant influence on dinoflagellate community structure when excluding effects of the spatial predictor (*p* < 0.05). Conversely, when excluding the influence of environmental predictors, the spatial predictor only explained 0.50% of the total variation and did not significantly influence the dinoflagellate community structure (*p* > 0.05, Fig. 5B).

## Discussion

### Geographical distribution of dinoflagellate communities

In the present study, only two environmental predictors (NO_3_-N and TDS) significantly influenced the geographical distribution of dinoflagellate communities along the Songhua River. After variance partitioning, the significant influence still remained, further highlighting the importance of these two environmental factors in shaping distribution patterns of dinoflagellate communities along the Songhua River. In general, NO_3_-N is essential for the growth and proliferation of plankton and has been reported with strong correlations with dinoflagellate abundance (Lafrancois et al., 2004). As expected, our results highlight the importance of NO_3_-N in affecting the variation of dinoflagellate abundance along the Songhua River. In contrast, TDS can have toxic effects on plankton (Ivanova & Kazantseva, 2006). Thus, the varied TDS concentration levels among sites can lead to different abundances of dinoflagellates (e.g., low abundance at site S10 with high TDS; Fig. 5A), further leading to geographical variation of communities.

However, both environmental and spatial predictors only explain 6.1% of the total variation, indicating that some unquantifiable variables play stronger roles in structuring the spatial distribution of dinoflagellate communities. These observations suggest that stochastic processes, such as temporal colonization and extinction events, should be additional factors in structuring dinoflagellate communities along the Songhua River (Grönros et al., 2013; Heino et al., 2015). Temporal colonization and extinction events have been frequently observed in ponds, especially temporary ponds where “strong small island effects” and “elements of chance” are expected to play key roles in structuring communities (Heino et al., 2015; Oertli et al., 2010). However, rapid extinction and colonization events are rarely observed in running water systems in general, as excessive dispersal can homogenize biological community structure in rivers and streams (Heino et al., 2015). Since we collected samples along the Songhua River during the wet season when it rained frequently and parts of waters may derive from basin ponds, the sampled dinoflagellate communities, or parts of the sampled communities, may derive from ponds. Consequently, only a small proportion of community variation could be explained by both environmental and spatial factors. Indeed, the small proportion of community variation explained by environmental and spatial factors have been commonly observed in aquatic ecosystems (Beisner et al., 2006; Devercelli et al., 2016). For example, one study in a lake ecosystem showed that only 8% of the variation of phytoplankton communities was explained by spatial factors and measured environmental parameters, and the authors argued that one-time sampling of phytoplankton communities and the absence of some important environmental variables (e.g., disturbance frequency) were the major reasons for this finding (Beisner et al., 2006). A study at fine geographical scale of a river floodplain showed that less than 17.8% of phytoplankton community variation was explained by environmental and spatial variables, and the authors suggested that random dispersion, ecological drift, and priority effects were important ecological processes responsible for phytoplankton meta-communities (Devercelli et al., 2016). Collectively, the low explanation power of environmental and spatial factors in this study may be attributed to four factors: (I) temporal colonization and extinction due to rainfall; (II) unmeasured environmental factors, such as relative light intensity and disturbance frequency, where the former is essential for the reproduction and growth of dinoflagellates and the latter has been shown to structure phytoplankton communities (Beisner, 2001); (III) higher trophic level predators, such as zooplankton, were not considered in this study; the abundance and taxa of predators can make significant influence on the prey composition and abundance variation (Kozak, Goldyn & Dondajewska, 2015); (IV) although the metabarcoding method shows robust power for diversity assessments of various communities (Zhan et al., 2014; Xiong et al., 2017; Yang et al., 2018), this method still cannot completely quantify the abundance of community composition (Sun et al., 2015), especially for taxonomic groups with large size variation. The poor relationship between sequence abundance based on molecular methods and real species abundance (Godhe et al., 2008) may disturb statistical analyses considerably for the exploration of complex interactions between organisms and environments.

Two genera, including *Ceratium* and *Unruhdinium*, were identified with high relative abundance along the Songhua River. Some species of *Ceratium*, such as *Ceratium hirundinella* and *Ceratium furcoides*, can form algae blooms (Van Ginkel, Hohls & Vermaak, 2001; Cassol et al., 2014). As such algae blooms may kill fish and lead to mass economic losses, as seen in Japan (Taylor, Fukuyo & Larson, 1995), harmful algae should be closely monitored for a better understanding of their population dynamics to manage economic and ecological issues along the Songhua River. Blooms of *Ceratium* spp. have been reported as the consequences of environmental changes, such as fluctuations of nutrient levels, temperature, and dissolved oxygen concentration (Periotoo et al., 2007; da Silva et al., 2012). Specifically, *Ceratium* spp. blooms were reported with relation to the increase of eutrophic levels in many rivers and lakes such as Furnas Reservoirs, Brazil (da Silva et al., 2012); however, related studies reported that the density of *Ceratium* spp. tended to become high under mesotrophic conditions (Periotoo et al., 2007). Thus, the relationship between trophic level and *Ceratium* spp. abundance was still unclear. Our results demonstrated that the relative abundance of *Ceratium* spp. varied largely among sites along the Songhua River. Although the RDA showed that the environmental predictors (NO_3_-N and TDS) should be responsible for the variation, the lower explanatory extent (<6.1%) made it difficult to infer a conclusion on the determinants of *Ceratium* spp. Hence, a deeper investigation covering different seasons is largely required to further recover the population fluctuation dynamics of *Ceratium*.

### The relative roles of species sorting and dispersal

The selected environmental factors (NO_3_-N and TDS) significantly explained only 5.85% of the total variation of dinoflagellate communities when the influence of watercourse distance was excluded. Interestingly, the dispersal predictor (watercourse distance) did not significantly influence dinoflagellate communities when excluding the influence of the environmental factors. The results obtained here are consistent with those in several other river systems such as Parana River floodplain (Devercelli et al., 2016) and Jiulong River (Isabwe et al., 2018), whereas opposite findings have been frequently observed (Datry et al., 2016; Heino et al., 2015). Usually, the inconsistent results may derive from whether there are significant environmental gradients in river systems. The Songhua River flows through three types of regions with different levels of pollutants such as NO_3_-N. Similar environmental gradients were also observed in Jiulong River (Isabwe et al., 2018) and Chaobai River (Xiong et al., 2017). The strong environmental gradients provide preconditions for species sorting, where species only occur at favorable environments. However, we cannot rule out a possible reason of discrepancy caused by the difference of communities (e.g., dinoflagellate versus phytoplankton). Since the relative contribution of species sorting and dispersal also depends on the taxonomic groups with diverse dispersal abilities and life histories (Beisner et al., 2006; Devercelli et al., 2016; Isabwe et al., 2018; Lin et al., 2014; Xiong et al., 2017), further studies on different taxonomic groups should be performed to verify whether the relative contribution of species sorting and dispersal differ among taxonomic groups.

## Conclusions

In summary, we used a metabarcoding-based approach to analyze the geographical distribution of dinoflagellate communities and identified factors responsible for the observed patterns. The minor proportion (6.1%) of community variation explained by environmental and spatial predictors indicates that additional stochastic processes, such as temporal extinction and colonization events, may play crucial roles in structuring dinoflagellate communities along the Songhua River during the wet season. The dissimilarity of dinoflagellate communities at the intra-group level was significantly greater than that at the inter-group level, suggesting in addition to two quantifiable processes (species sorting and dispersal), more complex processes should be involved in determining the community structure. Our study suggests that deeper investigations covering different seasons are required to understand the causes and consequences of geographical distribution of dinoflagellate communities and causative factors for the observed patterns on river ecosystems. Such information is crucial for both ecological surveys and conservation/management of biodiversity in different habitats.

##  Supplemental Information

10.7717/peerj.6733/supp-1 Figure S1 Information content at each nucleotide position for universal primer matching (Uni18S, Uni18SR, see Material and methods section). The plot is based on partial aligned small subunit ribosomal DNA (18S) sequences of selected dinoflagellate sequence

10.7717/peerj.6733/supp-2 Figure S2 Rarefaction curves of sequence reads from 23 samples along the Songhua River

10.7717/peerj.6733/supp-3 Figure S3 Dendrogram based on a Bray-Curtis dissimilarity of dinoflagellate communities along the Songhua River

10.7717/peerj.6733/supp-4 Supplemental Information 1 Tables S1-S4

## Additional Information and Declarations

### DNA Deposition

The following information was supplied regarding the deposition of DNA sequences: The sequencing data is available at NCBI (SRA accession no. SRP151183 and Bioproject PRJNA477641).

### Data Availability

The following information was supplied regarding data availability: The raw measurements are available in the Supplemental Files.
